# Earthquake Brain: Altered Recognition and Misclassification of Facial Expressions Are Related to Trauma Exposure but Not Posttraumatic Stress Disorder

**DOI:** 10.3389/fpsyt.2017.00278

**Published:** 2017-12-12

**Authors:** Caroline J. Bell, Helen C. Colhoun, Chris M. Frampton, Katie M. Douglas, Virginia V. W. McIntosh, Frances A. Carter, Jennifer Jordan, Janet D. Carter, Rebekah A. Smith, Leila M. A. Marie, Alex Loughlin, Richard J. Porter

**Affiliations:** ^1^Department of Psychological Medicine, University of Otago, Christchurch, New Zealand; ^2^Canterbury District Health Board, Christchurch, New Zealand; ^3^Department of Psychology, University of Canterbury, Christchurch, New Zealand

**Keywords:** faces, posttraumatic stress disorder, trauma-exposed controls, earthquakes, facial recognition

## Abstract

**Objectives:**

The study investigated facial expression recognition (FER) in posttraumatic stress disorder (PTSD) caused by exposure to earthquakes, and in particular whether people with this condition showed a bias toward interpreting facial expressions as threat-related emotions (i.e., as anger, fear, or disgust). The study included a trauma-exposed control group who had been similarly exposed to the earthquakes but had not developed PTSD. We hypothesized that individuals with PTSD would have increased sensitivity to threat-related facial emotions compared with the trauma-exposed control group. This would be shown by increased accuracy in recognition of threat-related emotions and the misinterpretation of neutral expressions to these emotions (i.e., misidentifying them as anger, fear, or disgust). The availability of a group of healthy controls from a previous study who had been tested on a similar task before the earthquakes allowed a further non-exposed comparison.

**Method:**

Twenty-eight individuals with PTSD (71% female, mean age 42.8 years) and 89 earthquake-exposed controls (66% female, mean age 50.1 years) completed an FER task, which featured six basic emotions. Further comparisons were made with 50 non-exposed controls (64% female, mean age 38.5 years) who had been tested before the earthquakes.

**Results:**

There was no difference in sensitivity to threat-related facial expressions (as measured by accuracy in recognition of threat-related facial expressions and the misinterpretation of neutral expressions as threatening) in individuals with PTSD compared with similarly earthquake-exposed controls. Supplementary comparison with an historical, non-exposed control group showed that both earthquake-exposed groups had increased accuracy for the identification of all facial emotions and showed a bias in the misclassification of neutral facial expressions to the threat-related emotions of anger and disgust.

**Conclusion:**

These findings suggest that it is exposure to earthquakes and repeated aftershocks, rather than the presence of PTSD that affects FER accuracy and misinterpretation. The importance of these biases in both PTSD and trauma-exposed controls needs further exploration and is an area for future research.

## Introduction

Posttraumatic stress disorder (PTSD) is a debilitating condition that develops in a significant minority of people after exposure to a traumatic event. There has been considerable interest in understanding the psychology and neuroscience of the condition, and in particular why only some people develop the disorder after a traumatic event whereas others, exposed to the same event do not (1).

Clinically individuals with PTSD often complain of impairments in emotional experience. These include reports of intense emotional reactions when exposed to reminders of their trauma suggesting emotional hyperresponsivity. They can also report emotional numbing (2) and alexithymia (difficulty labeling emotions) (3) suggesting emotional hyporesponsivity. Several models have been proposed to explain these diverse emotional responses in PTSD (2, 4).

Studies in PTSD have consistently reported attentional biases toward threat-related stimuli [from, e.g., performance on the modified Stroop (5), dot-probe tasks, and eye tracking (6)]. Psychological models of PTSD explain the role these biases have in perpetuating symptoms of the disorder (7).

Neurocircuitry models (8) have suggested that maladaptive top-down modulation of the amygdala by the medial prefrontal cortex results in a hyperactive fear network disengaged from upstream modulatory restraint by the prefrontal circuit (9–11). Recent connectivity studies have similarly reported that compared with trauma-exposed controls, patients with PTSD have weaker connectivity between the medial prefrontal cortex and amygdala and hippocampus (12). This hypersensitive fear network may underpin the biases in information processing found in PTSD (13), and in particular attention biases to trauma-related stimuli (14).

Interestingly recent neuroimaging studies comparing combat-exposed soldiers (with and without PTSD) and non-exposed controls have reported that both combat-exposed groups showed greater accuracy for threat-related stimuli (15). These findings suggest that exposure to trauma may in itself result in changes to attentional biases which are adaptive in the context of danger.

The ability to identify and interpret facial expressions is crucial for normal interpersonal relationships and for evaluating situations (16). Facial expressions signal the emotional states of others and influence the production and regulation of affective states in response to these signals (17). Studies of facial expression recognition (FER) in PTSD have reported mixed findings to date. Individuals with PTSD compared with combat-exposed controls have been found to have decreased accuracy and sensitivity of FER for fear, sadness (18) and anger (19). There have also been reports of ambiguous images being interpreted in a threatening way by combat-exposed soldiers (with PTSD or other trauma-related diagnoses) compared with non-exposed controls (20). These studies have all been in individuals exposed to combat where the use of FER may have represented a more specific trauma trigger, because the trauma in these cases related to interpersonal violence. Facial expressions have also been used as a probe of amygdala responsivity (21), with studies in PTSD reporting enhanced amygdala and diminished medial prefrontal cortical responses to threat-related facial expressions of fear and anger (9, 10, 22).

Over 2010–2011 Canterbury, New Zealand, experienced four major earthquakes (moment magnitude scale *M*_W_ > 6.0) and thousands of aftershocks, resulting in major damage throughout the city, 185 deaths, and thousands of injuries (23). This led to a large number of people being exposed to similar traumatic events and, therefore, created the opportunity to examine people with similar earthquake exposure who did or did not develop PTSD.

The aim of this study was to investigate FER in patients with PTSD compared with controls who had also been exposed to earthquakes. In particular, this task was used as a probe of general threat responsivity, by examining whether individuals with PTSD showed a bias toward threatening facial expressions. It was hypothesized that individuals with PTSD would have increased sensitivity to threat-related facial expressions compared with earthquake-exposed controls. This would be shown by increased accuracy in recognition of threat-related facial expressions (anger, fear, and disgust) and the misinterpretation of neutral expressions as threatening (misidentifying them as anger, fear, or disgust).

There has been increasing interest in examining brain changes as a result of exposure to trauma *per se* (24, 25). As a supplementary analysis it was decided to extend the comparison using historical data that had been collected from a previous study in a group of healthy individuals immediately before the earthquake sequence. This created two control groups, one that had been trauma-exposed and the other who had not been exposed to the earthquakes and allowed examination of the relative effects of exposure to the trauma of earthquakes and of a PTSD diagnosis. We hypothesized that exposure to trauma would result in increased sensitivity to threat-related facial expressions in both earthquake-exposed groups (PTSD and exposed controls) compared with non-exposed controls.

## Materials and Methods

### Participants

#### Earthquake-Exposed PTSD Group

This group was Canterbury residents with PTSD (*n* = 28) who had been referred for treatment to a Specialist Mental Health Service for members of the community with earthquake-related PTSD. These patients had a full clinical psychiatric assessment by an experienced clinician (Frances A. Carter, Helen C. Colhoun, Jennifer Jordan, Virginia V. W. McIntosh, and Caroline J. Bell) confirming the diagnosis before beginning treatment. Participants were recruited between February 2013 and April 2015 (which was between 2 and 4 years after the most devastating February 2011 earthquake).

#### Earthquake-Exposed Control Group

This group were Canterbury residents who self-identified as resilient, i.e., coping well, despite moderate to high exposure to earthquake-related events (such as physical injury or illness, death of a loved one, witnessing falling buildings, seeing bodies, property loss, income loss, or problems with housing caused by earthquake-related events) (*n* = 89). They were recruited in response to articles, opinion pieces, and community notices in local newspapers, and *via* word of mouth over the course of 13 months, from January 2013 to February 2014. These participants had a face-to-face assessment (by Rebekah A. Smith, Leila M. A. Marie, and Alex Loughlin) and completed diagnostic and self-report questionnaires, to confirm that they had no earthquake-related psychiatric diagnoses and had not received any earthquake-related counseling.

#### Supplementary Analysis—Comparison with Non-Exposed Control Group

This control group (*n* = 50) was from an historical study investigating neuropsychological functioning in severe depression (26). These participants were all psychologically healthy individuals without a personal or family history of mental illness. They were recruited through advertisements in local newspapers and flyers in public places from the general population in Canterbury before the earthquakes between June 2007 and March 2009 and thus represent a non-exposed comparison group.

### FER Task

Participants performed a modified version of the FER task (27), which was presented on a computer using E-Prime software (E-Prime^®^ 2.0). Faces displaying five basic emotions (anger, happy, sad, fearful, and disgusted expressions) were randomly presented successively on a computer screen for 500 ms, followed immediately by a blank screen. The faces had been morphed into varying intensities of each emotion from 50% emotion (50% emotion and 50% neutral) to 100% emotion, in 10% steps. Neutral facial expressions (0% emotion) were also presented. Participants were instructed to identify the emotions and to press one of six labeled buttons on a response pad (five emotions and neutral) as quickly and as accurately as possible.

The historical, non-exposed group completed a slightly different version of the FER task; they were presented with 96 faces (16 faces for each emotion and 16 neutral faces) whereas the PTSD and earthquake-exposed control groups were presented with 150 faces (20 faces of each emotion and 50 neutral faces). The decision to increase the proportion of neutral facial expressions in the earthquake-exposed groups was because of our previous work, which suggested that misinterpretation of neutral expressions was more powerful than emotion recognition accuracy in identifying negative or threat-related biases (28, 29). For this reason, the analysis was repeated only including the first 96 trials (out of 150) from the PTSD and earthquake-exposed control groups.

### Procedure

Assessments took place at a University Clinical Research Unit in Christchurch, New Zealand. Participants completed a diagnostic interview with the Mini-International Neuropsychiatric Interview [MINI (30)] and self-report demographic measures. The earthquake-exposed groups (PTSD and earthquake-exposed controls) also completed other clinical measures. These included the PTSD Checklist (PCL-S) (31), Depression, Anxiety, and Stress Scale (DASS) (32), Connor–Davidson Resilience Scale (CDRS) (33), Social Adjustment Scale (34), a Visual Analog Scale rating anxiety on the test day using a 10 cm scale (0 = no anxiety to 5 = severe anxiety), Traumatic Exposure Severity Scale (TESS) (35), and the Life Events Scale (LES), adapted from the Crisis in Family Systems (CRSYS) (36).

The PCL-S assesses current (past month) symptoms of PTSD in relation to an identified stressful experience (earthquakes in this study). It consists of 17 items which correspond to DSM-IV symptoms and asks how often the person has been bothered by each symptom coded from 1 to 5 (1 = not at all, through to 5 = extremely). The total symptom severity score ranges from 17 to 85 with scores above 44 being in the clinical range (37).

The DASS-21 measures 21 symptoms over the past week; seven items each for depression, anxiety, and stress rated from 0 to 3 (0 = did not apply to me, through to 3 = applied to me very much or most of the time) (38). The DASS-21 scores (doubled to be comparable to the DASS-42) indicate overall severity of symptomatology and give subscale totals for depression, anxiety, and stress. For the depression subscale, normal-mild has been defined as a score of 0–13 and >14 as moderate-extreme. For the anxiety subscale, normal-mild has been defined as a score of 0–9 and >10 as moderate-extreme. For the stress subscale, normal-mild has been defined as a score of 0–18 and >19 as moderate-extreme (32).

The CDRS assesses resilience over the past month. It consists of 25 items rated from 0 to 4 (0 = not true at all, through to 4 = true nearly all the time). The scores are summed and range from 0 to 100 with higher scores reflecting greater resilience.

The SAS assesses social functioning over the past 2 weeks. It consists of 45 items each rated from 1 to 5 with higher scores indicating greater impairment. The final score is derived by summing the scores and dividing by the number of items and ranges between 1 and 5.

The TESS measures the number of earthquake-related stressors and the distress experienced in relation to these. Examples of items included: were you or your loved ones injured?, did you see falling buildings? and was your home damaged? It consists of 24 items, and participants are asked whether they had experienced the stressor (yes/no) and if yes, how distressing this had been on a scale where 1 = not at all and 5 = extremely. The TESS number of exposures and distress scores are summed.

The LES measures the number of contemporary life stressors and how difficult these were within the last 5 years and the last 6 months. Examples of items included: did your income increase or decrease? did you have legal problems? and did you marry or divorce? It consists of 63 items in 11 content domains (financial, legal, career, stability in relationships, safety in the home, safety in the community, medical issues pertaining to respondent, medical issues pertaining to others, housing problems, difficulty with authority, and prejudice). Participants are asked whether they had experienced the life event (yes/no) and, if yes, how difficult his had been on a scale where 1 = not at all and 5 = extremely. The LES number of events and difficulty scores are summed.

The study was approved by the National Health and Disability Ethics Committee URA/12/03/11, and written informed consent was given before participation in the studies.

### Statistical Analyses

Statistical analyses were conducted using the Statistical Package for Social Sciences version 23 for Windows. Demographic and clinical data were summarized using standard descriptive statistics including means, SEs, ranges, frequencies, and percentages as appropriate. Comparison of demographic and clinical variables between the PTSD and earthquake-exposed controls used ANOVA and chi-square tests. Comparisons of FER accuracy and interpretation bias (the emotion incorrectly ascribed to a neutral face) was conducted using ANCOVA with group as the between-participant factor and age, gender, years of education, anxiety level on test day, depression (depression subscale score of the DASS) as covariates to adjust for baseline clinical features known to impact on FER functioning.

For supplementary analyses, the same comparisons were extended to include the non-exposed controls with similar comparisons using ANCOVA adjusting for age, gender, and years of education. Clinical variables were not included as covariates as the non-exposed controls did not complete clinical questionnaires.

A two-tailed *p*-value < 0.05 was taken to indicate statistical significance.

## Results

### Demographic and Clinical Measures

Table 1 shows that there was a similar proportion of males and females in the two earthquake-exposed groups (PTSD group 71% female; earthquake-exposed control group 66% female), and that the earthquake-exposed controls were significantly older and had more years of education than the PTSD group (PTSD group mean age 42.8 years; earthquake-exposed controls mean age 50.1 years). As expected, the PTSD group had markedly increased rates of current PTSD, depression, other anxiety disorders and antidepressant use. Of the PTSD group, eight participants (32%) had a previous lifetime diagnosis of PTSD related to earlier traumatic event exposure before the earthquakes. This PTSD was in full, sustained remission at the time of the earthquakes in seven of these eight participants; and in one participant this PTSD was in partial remission. None of the earthquake-exposed controls had a current or previous diagnosis of PTSD. The one individual in the earthquake-exposed control group with current depression and two with current panic disorder had had these disorders before the start of the earthquakes. Table 1 shows the expected clinical differences between the PTSD and earthquake-exposed controls as measured by symptoms of PTSD [Posttraumatic Check List (PCL)], DASS, resilience (CDRS), general functioning (SAS), and ratings of anxiety on the test day. The PCL scores are significantly different between the PTSD and earthquake-exposed groups with the scores having no overlap between the groups; the PCL mean score in the PTSD group of 53.5 was within the clinical range (>44) whereas the mean score in the earthquake-exposed group was 21.8, which is close to the minimum possible score of 17. However, the two groups reported experiencing similar levels of distress from earthquake exposure (from the TESS) and from life events (from the LES).

**Table 1:** Comparison of demographic and clinical variables between posttraumatic stress disorder (PTSD) group, earthquake-exposed controls, and non-exposed controls (in italics).

	PTSD group (*N* = 28)	Earthquake-exposed control group (*N* = 89)	Non-exposed control group (*N* = 50)	*p*-Value
**Gender**				
Percentage (number) female	71 (20)	66 (58)	*64* (32)	NS

**Age**				
Mean years (SD)	42.8 (12.8)	50.1 (11.4)	*38.5 (9.8)*	<0.001*

**Years of secondary/tertiary education**				
Mean (SD)	4.9 (2.1)	7.1 (2.7)	*6.1 (1.9)*	<0.001*

**Time from February 22, 2011 earthquake (days)**				
Mean (SD)	1,065 (218)	767 (98)	*NA*	<0.001*

**Current diagnosis on Mini-International Neuropsychiatric Interview**				
% (number) PTSD	100 (28)	0 (0)	*0 (0)*	<0.001**
% (number) Depression	79 (22)	1 (1)	*0 (0)*	<0.001**
% (number) Social phobia	36 (10)	0 (0)	*0 (0)*	<0.001**
% (number) Panic disorder	54 (15)	2 (2)	*0 (0)*	<0.001**
% (number) GAD	50 (14)	0 (0)	*0 (0)*	<0.001**
% (number) OCD	11 (3)	0 (0)	*0 (0)*	<0.001**

**On antidepressant medication**				
% (number)	58 (16)	5 (4)	*0 (0)*	<0.001**

**Posttraumatic Check List**				
Mean (SD)				
Total score (*range*)	56 (9.6) (*44–72*)	22 (4.8) (*17–36*)	*NA*	<0.001*
Re-experiencing	16.1 (4.6)	6.1 (1.4)	*NA*	<0.001*
Avoidance/numbing	20.3 (5.8)	8.7 (2.5)	*NA*	<0.001*
Hyperarousal	17.1 (4.3)	7.0 (2.3)	*NA*	<0.001*

**Depression Anxiety Stress Scale 21 (DASS)**				
% Depression (moderate-extreme)	78	1	*NA*	<0.001**
% Anxiety (moderate-extreme)	89	1	*NA*	<0.001**
% Stress (moderate-extreme)	75	1	*NA*	<0.001**

**Connor–Davidson Resilience Scale**				
Mean (SD)	47 (13.5)	75 (12.0)	*NA*	<0.001*

**Social Adjustment Scale (SAS)**				
Mean (SD)	2.5 (0.4)	1.7 (0.3)	*NA*	<0.001*

**Traumatic Exposure Severity Scale (TESS)**				
Mean number of exposures (SD)	23.6 (15.4)	15.8 (10.9)	*NA*	0.03*
Mean distress (SD)	6.5 (2.7)	5.3 (2.5)	*NA*	NS

**Life Events Scale**				
Mean number (last 5 years) (SD)	14.1 (5.9)	8.4 (4.7)	*NA*	<0.001*
Mean number (last 6 months) (SD)	6.4 (3.9)	3.5 (2.7)	*NA*	0.01*
Mean difficulty (last 5 years) (SD)	2.2 (1.4)	2.1 (0.7)	*NA*	NS

**Anxiety on test day**				
Mean visual Analog Scale (0–5) (SD)	2.5 (1.7)	1.0 (1.2)	*NA*	<0.001*

*NS, not significant*.

*Significant ANOVA.

**Significant chi-square test.

Testing days for the two groups occurred some considerable time after the most devastating February 2011 earthquake; mean of 755 (SD 108) days for the earthquake-exposed controls and 1,053 (SD 201) days for the PTSD group. The difference between the two groups was due to PTSD patients being recruited as they were referred for treatment, whereas the earthquake-exposed controls were a research cohort recruited over a specific timeframe.

### FER Task

#### Accuracy

This was defined as the correct identification of facial expressions of emotions at each intensity level. Accuracy scores for each facial expression of emotion were averaged across the four actors portraying the same facial emotion. Scores were normally distributed. Repeated measures ANCOVA showed no significant effect of group for accuracy of any facial expressions; neutral *F*(1, 115) = 0.52, *p* = 0.47; anger *F*(1, 115) = 0.22, *p* = 0.64; happy *F*(1, 115) = 0.00, *p* = 0.99; sad *F*(1, 115) = 0.95, *p* = 0.33; fearful *F*(1, 115) = 2.26, *p* = 0.14; disgust *F*(1, 115) = 0.40, *p* = 0.53; and overall accuracy *F*(1, 115) = 0.41, *p* = 0.53.

#### Misinterpretation of Neutral Expressions

This was defined as the percentage of neutral expressions misclassified as an emotion. Scores were normally distributed. Repeated measures ANCOVA showed no significant effect of group for neutral expressions misclassified to any emotion; anger *F*(1, 113) = 0.004, *p* = 0.95; happy *F*(1, 113) = 0.12, *p* = 0.46; sad *F*(1, 113) = 0.12, *p* = 0.73; fearful *F*(1, 113) = 0.57, *p* = 0.45; and disgust *F*(1, 113) = 0.09, *p* = 0.77.

#### Reaction Time

Reaction time was the time taken to press one of the six labeled buttons (five emotions and neutral) on a response pad when completing the FER task. Reaction times for each facial expression of emotion were averaged across the four actors portraying the same facial emotion. Scores were normally distributed. Repeated measures ANCOVA showed no significant effect of group for any of the facial expressions; neutral *F*(1, 115) = 2.65, *p* = 0.717; happy *F*(1, 115) = 5.72, *p* = 0.644; anger *F*(1, 115) = 0.17, *p* = 0.721; sad *F*(1, 115) = 3.63, *p* = 0.993; fearful *F*(1, 115) = 4.79, *p* = 0.370; and disgust *F*(1, 115) = 9.89, *p* = 0.781.

#### Supplementary Analysis—Comparison of PTSD Group, Earthquake-Exposed Controls, and Non-Exposed Control Groups

Demographic and clinical variables for the non-exposed control groups are shown in Table 1. This group had a similar proportion of males and females as the two earthquake-exposed groups, i.e., 64% female. The mean age of this group was 38.5 years, which was significantly younger than the earthquake-exposed controls. This group had no current or lifetime diagnosis of any psychiatric disorder.

Analysis of performance on the FER measures by ANCOVA comparing PTSD, earthquake-exposed and non-exposed control groups with age, gender, and years of education as covariates are presented in Table 2. Clinical variables of depression and anxiety on the test day were not included as covariates in this analysis because the non-exposed control group did not complete clinical questionnaires.

**Table 2:** ANCOVA showing group effects of facial expression recognition in posttraumatic stress disorder (PTSD), earthquake-exposed controls, and the non-exposed controls (in italics).

	PTSD group (*N* = 28), mean (SE)	Earthquake-exposed control group (*N* = 89), mean (SE)	Non-exposed control group (*N* = 50), mean (SE)	*F*	*p*-Value
**% Accuracy**					

Neutral	73.3 (3.5)	70.3 (2.1)	*60.0 (2.7)*	6.0	0.003*
Anger	56.0 (3.3)	60.0 (2.0)	*40.3 (2.6)*	17.9	<0.001*
Happy	92.8 (1.7)	93.0 (1.0)	*84.4 (1.3)*	13.6	<0.001*
Sad	55.2 (3.4)	63.8 (2.0)	*20.7 (2.6)*	83.4	<0.001*
Fearful	78.7 (3.3)	85.2 (2.0)	*53.1 (2.6)*	48.3	<0.001*
Disgust	67.1 (4.2)	70.1 (2.5)	*40.0 (3.1)*	26.9	<0.001*

**% Misinterpretation neutral expressions**					

To anger	38.6 (4.6)	40.1 (2.8)	*11.1 (3.5)*	21.7	<0.001*
To happy	14.2 (3.9)	10.3 (2.3)	*54.9 (3.0)*	70.5	<0.001*
To sad	27.1 (4.3)	27.7 (2.6)	*24.3 (3.3)*	0.3	0.721
To fearful	8.8 (2.8)	10.7 (1.7)	*7.1 (2.2)*	0.8	0.449
To disgust	11.4 (3.0)	11.2 (1.8)	*2.1 (2.3)*	5.4	0.005*

**Reaction time**					

Neutral	2,047 (111)	1,783 (67)	*1,697 (86)*	3.4	0.037*
Anger	2,532 (141)	2,126 (85)	*2,461 (109)*	4.4	0.014*
Happy	1,671 (222)	1,541 (222)	*1,297 (172)*	1.0	0.354
Sad	2,329 (101)	1,942 (60)	*1,641 (78)*	15.4	<0.001*
Fearful	2,328 (128)	1,870 (77)	*2,235 (100)*	6.5	0.002*
Disgust	2,278 (125)	1,809 (75)	*2,187 (970)*	7.2	0.001*

*Means are estimated means with gender, years of education, and age as covariates*.

*Significant ANCOVA.

### FER Task

#### Accuracy

**Figure 1:**
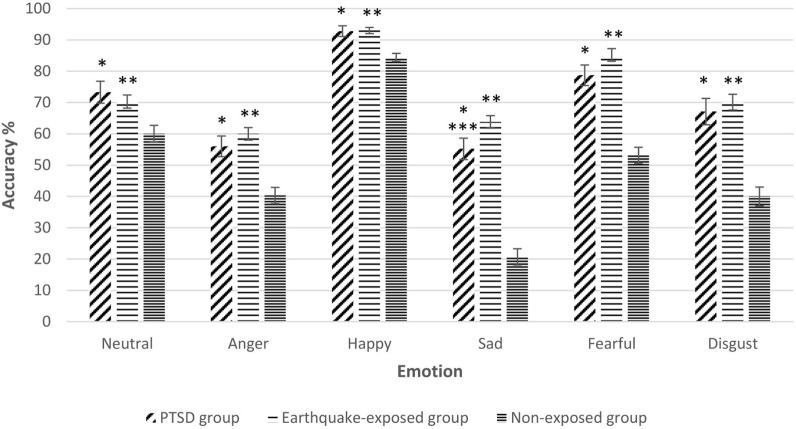
Gender, years of education, and age-adjusted recognition accuracy (mean and SEM) for the five expressions of emotion and neutral expressions on the facial expression recognition task in the three groups. Pairwise comparisons significantly different *p* < 0.05. *Posttraumatic stress disorder (PTSD) group significantly different from non-exposed group. **Earthquake-exposed group significantly different from non-exposed group. ***PTSD group significantly different from earthquake-exposed group.

Repeated measures ANCOVA including the non-exposed healthy controls showed a significant effect of group for accuracy of all facial expressions. Figure 1 shows the mean percentage accuracy of FER for each emotion for the three groups adjusted for gender, years of education, and age (which were significantly different among the groups). Pairwise comparisons showed that both earthquake-exposed groups (PTSD and earthquake-exposed controls) were significantly more accurate in recognizing all facial emotions compared with the non-exposed control group (PTSD comparison with non-exposed; neutral (*p* = 0.003), anger (*p* < 0.001), happy (*p* < 0.001), sad (*p* < 0.001), fearful (*p* < 0.001), and disgust (*p* < 0.001); earthquake-exposed comparison with non-exposed; neutral (*p* < 0.001), anger (*p* < 0.001), happy (*p* < 0.001), sad (*p* < 0.001), fearful (*p* < 0.001), and disgust (*p* < 0.001)). Pairwise comparison between the PTSD and earthquake-exposed group showed that the PTSD group was significantly less accurate at identifying the emotion of sad (*p* = 0.029). The difference from the earlier analysis above comparing the PTSD and earthquake-exposed group is explained by the fact that this ANCOVA did not include clinical measures (such as of depression) as covariates.

The analysis was repeated only including the first 96 trials (out of 150) from the PTSD and earthquake-exposed groups to allow for the slightly different version of the FER task used in the non-exposed group which included only 96 faces. The results from this analysis were unchanged from those reported above, with the results remaining significant.

#### Misinterpretation of Neutral Expressions

**Figure 2:**
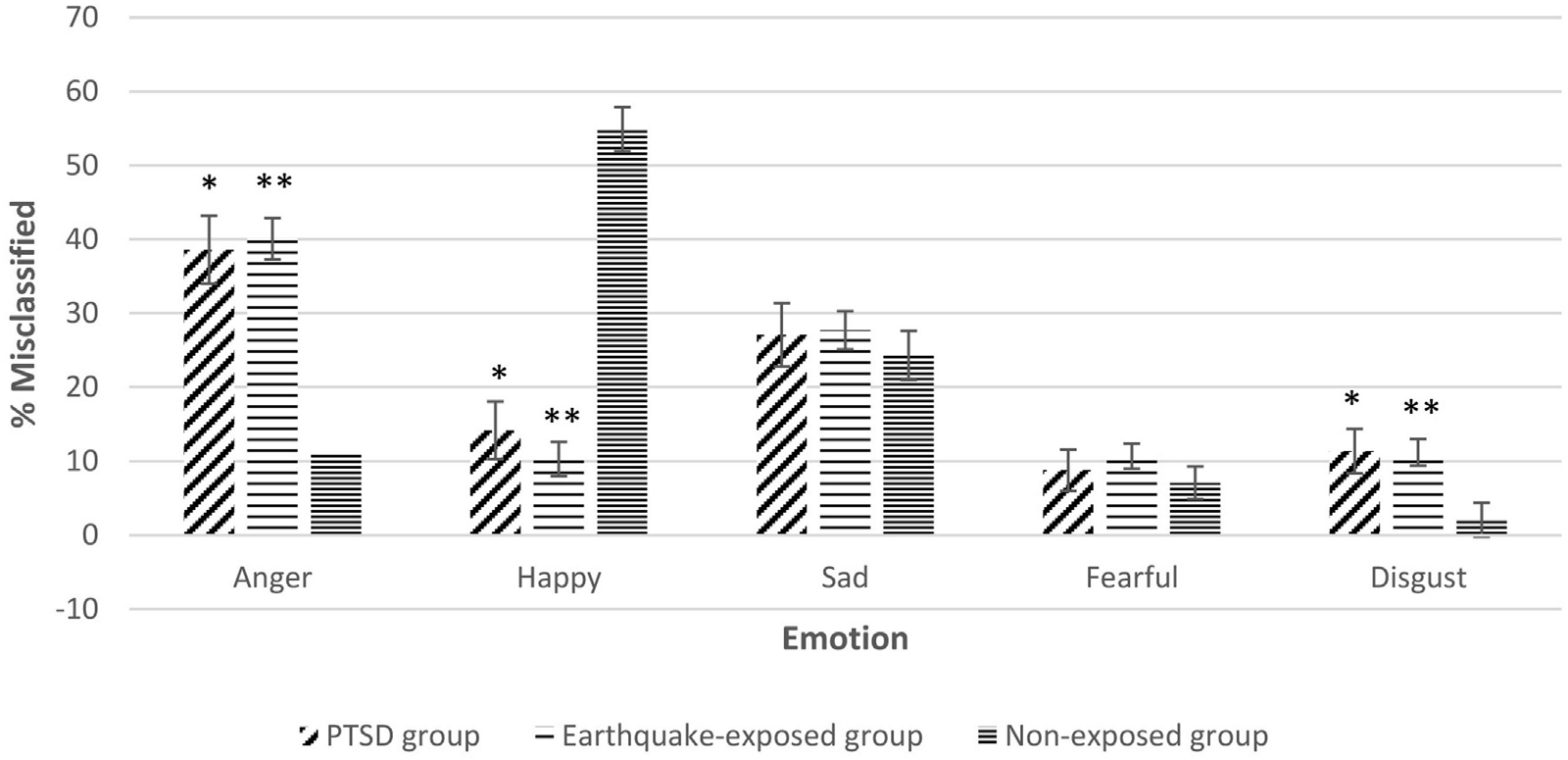
Gender, years of education, and age-adjusted misclassification of neutral expression to different emotions in the three groups. Pairwise comparisons significantly different *p* < 0.05. *Posttraumatic stress disorder (PTSD) group significantly different from non-exposed group. **Earthquake-exposed group significantly different from non-exposed group.

Repeated measures ANCOVA including the non-exposed healthy controls showed significant effect of group for misinterpretation of neutral expressions as anger, disgust, and happy. Figure 2 shows the misclassification of neutral expressions to different emotions for the three groups adjusted for gender, years of education, and age. Pairwise comparisons showed that the two earthquake-exposed groups (PTSD and earthquake-exposed controls) were significantly more likely to attribute the emotions of anger and disgust to neutral expressions compared with the non-exposed control group [PTSD comparison with non-exposed; anger (*p* < 0.001) and disgust (*p* = 0.012); earthquake-exposed comparison with non-exposed; anger (*p* < 0.001) and disgust (*p* = 0.003)]. The non-exposed group was significantly more likely to interpret neutral expressions as happy (*p* < 0.001).

#### Reaction Times

Repeated measures ANCOVA including the non-exposed healthy controls showed significant effect of group for reaction times to all emotions apart from happy. Pairwise comparisons showed that; the PTSD group was significantly slower than the non-exposed group for neutral (*p* < 0.001) and sad (*p* < 0.001) emotions; the earthquake-exposed group were slower than the non-exposed group for sad (*p* = 0.003), and faster for the emotions of anger (*p* = 0.02), fearful (*p* = 0.005) and disgust (*p* = 0.03); the PTSD group were slower than the earthquake-exposed group for neutral (*p* = 0.041), anger (*p* = 0.014), sad (*p* = 0.001), fearful (*p* = 0.002), and disgust (*p* = 0.001). As noted above, the difference from the earlier analysis comparing the PTSD and earthquake-exposed groups is explained by the fact that this ANCOVA did not include clinical measures (such as of depression) as covariates.

## Discussion

The aim of this study was to investigate FER in PTSD, and in particular to examine whether individuals with PTSD showed a bias toward identifying facial expressions as threatening (i.e., as anger, fear, or disgust). We hypothesized that compared with trauma-exposed controls, individuals with PTSD would have an increased sensitivity to threat-related facial expressions. This would be shown by increased accuracy in recognition of threat-related facial expressions (anger, fear, and disgust) and the misinterpretation of neutral expressions as threatening (i.e., misidentifying them as anger, fear, or disgust). Counter to our hypothesis we found no such differences.

As expected the PTSD group had significant PTSD (as evidenced by the MINI diagnostic interview and the self-report scores from the PCL), depression and other mental health disorders. The earthquake-exposed control group, who had self-identified as coping well, also had diagnostic and self-report scores reflecting this and confirming that they were a resilient group and not a sub-clinical sample. Interestingly neither of the groups had specifically greater increased ratings of hyperarousal symptoms from the PCL despite exposure to ongoing aftershocks and other stressors as a result of the earthquakes. Both groups reported similar levels of distress or difficulty from their experiences (from the TESS and the LES) but despite this the earthquake-exposed controls reported functioning well. Despite both groups having been exposed to stressful events it was only in the PTSD group that this had resulted in a significant impact on their mental health and functioning. Other authors have suggested that this different impact of traumatic events may be explained by differences in the appraisal of the risk associated with the experiences and as a result the fear response that is triggered (39).

Findings from this study show no difference in the accuracy of FER, misclassification of neutral expressions or reaction times in individuals with PTSD compared with earthquake-exposed controls. Other clinical studies of FER accuracy have reported mixed findings. One study in children who had been maltreated reported no differences between individuals with PTSD and trauma-exposed controls (40). Two other studies in war veterans, however, reported that those with PTSD had reduced accuracy for the facial expressions of fear, sadness (18), and anger (19), and suggested that this may explain reports of alexithymia often found in individuals with this disorder. Both studies, in contrast to the current report, involved significant interpersonal violence, which may be an important factor, particularly when considering possible increased sensitivity to identification of threat-related expressions. Although this study found no gender effects it is an important addition to the literature as it has had a high proportion of female participants whereas previous studies have been predominantly in males.

The results from the comparison with the non-exposed controls, which did not include measures of depression as covariates, showed some differences between the PTSD and earthquake-exposed groups in the accuracy in recognizing facial expressions of sad emotions and psychomotor speed. These differences were not strong and may represent Type 1 errors as a consequence of this additional testing. They may suggest an association with symptoms of depression rather than a direct effect of PTSD.

The extended comparison (in the supplementary analysis) with the use of two control groups, one which had been similarly earthquake-exposed and the other which had not been exposed, allowed the examination of contributions from exposure to earthquakes and a diagnosis of PTSD. Individuals with PTSD and earthquake-exposed controls showed increased accuracy for the identification of all facial emotions compared with non-exposed controls. They also showed a bias in the misclassification of neutral facial expressions to the threat-related emotions of anger and disgust.

The general increase in accuracy of FER across all emotions suggests possible diversion of resources away from other functions in favor of emotional processing possibly secondary to a degree of hypervigilance. Clearly it may be advantageous to be accurate in processing of others facial expressions when under threat. What is slightly surprising is that the increased accuracy is not restricted to threat-related expressions as we had hypothesized. Previously neuroimaging studies in PTSD related to combat have reported enhanced activation to threat-related facial expressions in PTSD compared with other facial emotions (10). It is possible that the difference in the findings may be explained by the prolonged exposure to aftershocks in the earthquake-exposed groups.

This study found, as has been previously reported, that healthy, non-exposed individuals tend to be more likely to misinterpret neutral expressions as happy than any other emotion (41). Our results show that this is reversed in those exposed to earthquakes who are less likely to misclassify to happy and more likely to misclassify to threat-related expressions. This suggests increased vigilance and a response bias for threat-related expressions. The exception is that there was no tendency in the traumatized groups to misclassify to the facial expression of fear. This may be explained by previous work, which has suggested that angry faces are thought to represent direct threat to a person whereas fearful faces represent indirect or non-ambiguous threat (42). The increased misclassification of neutral expressions to disgust may reflect increased insula hypersensitivity, which is also part of the threat detection brain network (43) as disgust recognition has been associated with this region.

The earthquake-exposed groups (both PTSD and earthquake-exposed controls) had slower reaction times than the non-exposed group for the non-threat-related emotion of sad possibly suggesting that both exposed groups were examining these non-threat emotions more closely and therefore having slower reaction times. Interestingly only the earthquake-exposed control group (and not the PTSD group) had faster reaction times than the non-exposed group to the threat-related emotions (anger, fear, fear, and disgust) suggesting hypervigilance and attentional bias to these emotions.

The fact that similar results were found in both earthquake-exposed groups (PTSD and earthquake-exposed controls) from FER measures of accuracy and misidentification suggests that it was earthquake (trauma) exposure itself (rather than the presence of PTSD) that affected FER performance in these groups. This may be due to living in an environment where threat and ongoing seismic activity were part of everyday life. It is important to note that over a 2-year period Canterbury experienced over 10,000 aftershocks and it may be that this resulted in biological changes in, e.g., the amygdala and residents being in a chronically hyperaroused state.

Studies comparing combat-exposed soldiers (with and without PTSD) and non-exposed controls have reported that both combat-exposed groups showed greater accuracy for threat-related stimuli (15) and a negative interpretation bias to ambiguous stimuli (20). These studies both involved interpersonal trauma exposure. It is interesting that the findings from this study report similar results even though the trauma of the earthquake was not a socially threatening event. It may be that there are even stronger differences after the exposure to interpersonal traumatic events, such as sexual assaults. Neuroimaging studies where the trauma was not earthquakes (i.e., related to combat and sexual assault) have found that in comparison with healthy controls, trauma-exposed controls and individuals with PTSD both showed greater amygdala activation (10, 44). However, these studies also report that trauma-exposed controls showed greater prefrontal connectivity compared with the PTSD group which the authors hypothesized may be associated with greater resilience (10). Similarly a recent neuroimaging study following an earthquake reported that compared with trauma-exposed controls (without PTSD), individuals with PTSD had weaker connectivity between the medial prefrontal cortex and amygdala and hippocampus (12). These authors suggest that individuals exposed to trauma who do not develop PTSD, activate more top-down modulatory areas while those who develop PTSD show less neural connectivity with emotion regulation centers (45).

There has been increasing interest in the long-term impacts of exposure to trauma on the brain. Animal studies have reported lasting effects of exposure to severe or prolonged stress with increased amygdala responsivity and reduced prefrontal cortical regulation (46). A recent study examining the long-term effects (41 months) of stress from exposure to earthquakes reported that compared with non-exposed controls, individuals exposed to earthquakes who did not have PTSD had greater gray matter density in prefrontal–limbic systems related to emotion regulation (24). The findings from this study, which was conducted 2–3 years after exposure to the earthquakes, would also suggest long-term impacts on the brain even in individuals who self-identify as resilient and coping well.

From a clinical perspective, it could be hypothesized that trauma exposure, particularly in an environment of ongoing threat as posed by the ongoing aftershock environment, required a state of hyperarousal to maintain safety. Similar high levels of hyperarousal have been reported in veterans without PTSD (25) The attentional biases found may well be adaptive in this context when there is advantage in attending to potential threat rather than underestimating it. However, although maintaining a constant state of vigilance is adaptive in unpredictable and dangerous situations, this tendency may be maladaptive if it continues long term and is likely to contribute to other symptoms such as disrupted sleep and difficulty concentrating (47).

Future research is needed to investigate whether hypersensitivity to threat was found in relation only to FER or generalized to other stimuli (48). It would also be interesting to examine whether these biases are trait or state phenomena and improve with time in the earthquake-exposed groups.

This study has the following limitations. There was a difference in the time of testing between the two earthquake-exposed groups. However, both were more than 2 years from the beginning of the earthquake sequence and it is unlikely that this would influence the results. There were also some limitations associated with the groups; individuals in the PTSD group although presenting clinically with PTSD as the primary diagnosis, had considerable comorbidity which limits what can be inferred as being related to a diagnosis of PTSD; and in comparison with the PTSD group, the earthquake-exposed controls were older and more educated. However, this was adjusted for statistically. A final issue is that of limited statistical power related to the size of the PTSD group. This may have resulted in type II errors in the comparison between the PTSD and control groups.

There are also some limitations associated specifically with the use of an historical sample as the non-exposed comparison group. The decision to do this was based on reports describing symptoms and brain changes as a result of exposure to trauma *per se* which meant that comparison with a non-exposed control group would provide important information. It would not have been possible to access a non-exposed group without going outside the region, due to the widespread impact of the disaster. Issues associated with the use of this additional comparison included some differences in the self-report scales and FER methodology with a greater number of trials (150) and more neutral expressions in the test battery for the two earthquake-exposed groups compared with the non-exposed controls. This may have allowed participants to habituate to testing and explain the finding of increased accuracy in the earthquake-exposed groups. For this reason the analysis was repeated only including the first 96 faces (as used in the non-exposed FER task) and similar results were found. We feel that it is unlikely that this methodological issue would affect the misidentification of specific emotions finding.

In conclusion the key finding from this study was that no differences were found in sensitivity to threat-related facial expressions (as measured by accuracy in recognition of threat-related facial expressions and the misinterpretation of neutral expressions as threatening) in individuals with PTSD compared with similarly earthquake-exposed controls. A supplementary comparison with a non-exposed control group showed that both earthquake-exposed groups had increased sensitivity to threat compared with the non-exposed controls. This offers preliminary evidence suggesting that it is earthquake (trauma) exposure itself, rather than the presence of PTSD that affects FER accuracy and misinterpretation. Although these findings require replication, it may be that trauma exposure, particularly in an environment of prolonged threat as posed by the ongoing earthquake and aftershock sequence, required a state of vigilance to maintain safety.

## Ethics Statement

The study was approved by the National Health and Disability Ethics Committee URA/12/03/11, and written informed consent was given before participation in the studies.

## Author Contributions

CB, HC, VM, FC, JJ, JC, RS, LM, AL, KD, and RP designed the study and wrote the protocol. CB and CF undertook the statistical analysis. CB, HC, and RP wrote the first draft of the manuscript. All the authors commented on the manuscript; contributed to and have approved the final manuscript.

## Conflict of Interest Statement

The authors declare that the research was conducted in the absence of any commercial or financial relationships that could be construed as a potential conflict of interest.
